# Regulation of Inflammation by Short Chain Fatty Acids

**DOI:** 10.3390/nu3100858

**Published:** 2011-10-14

**Authors:** Marco A.R. Vinolo, Hosana G. Rodrigues, Renato T. Nachbar, Rui Curi

**Affiliations:** Department of Physiology and Biophysics, Institute of Biomedical Sciences-ICB-I, Sao Paulo University, Brazil; Email: hosanagr@icb.usp.br (H.G.R.); nachbar@icb.usp.br (R.T.N.); ruicuri@icb.usp.br (R.C.)

**Keywords:** short chain fatty acids, inflammation, leukocytes, neutrophils, butyrate, propionate, acetate

## Abstract

The short chain fatty acids (SCFAs) acetate (C_2_), propionate (C_3_) and butyrate (C_4_) are the main metabolic products of anaerobic bacteria fermentation in the intestine. In addition to their important role as fuel for intestinal epithelial cells, SCFAs modulate different processes in the gastrointestinal (GI) tract such as electrolyte and water absorption. These fatty acids have been recognized as potential mediators involved in the effects of gut microbiota on intestinal immune function. SCFAs act on leukocytes and endothelial cells through at least two mechanisms: activation of GPCRs (GPR41 and GPR43) and inhibiton of histone deacetylase (HDAC). SCFAs regulate several leukocyte functions including production of cytokines (TNF-α, IL-2, IL-6 and IL-10), eicosanoids and chemokines (e.g., MCP-1 and CINC-2). The ability of leukocytes to migrate to the foci of inflammation and to destroy microbial pathogens also seems to be affected by the SCFAs. In this review, the latest research that describes how SCFAs regulate the inflammatory process is presented. The effects of these fatty acids on isolated cells (leukocytes, endothelial and intestinal epithelial cells) and, particularly, on the recruitment and activation of leukocytes are discussed. Therapeutic application of these fatty acids for the treatment of inflammatory pathologies is also highlighted.

## Abbreviations

ALI: acute lung injury; AT: adipose tissue; CINC-2: cytokine induce neutrophil chemoattractant-2; FAK: focal adhesion kinase; fMLP: *N*-formyl-Methionine-Leucine-Phenylalanine; GI: gastrointestinal; GPCR: G protein coupled receptors; GRO: growth-related oncogene; HAT: histone acetyltransferase; HDAC: histone deacetylase; HUVEC: human umbilical vein endothelial cells; IBD: inflammatory bowel disease; ICAM-1: inter-cellular adhesion molecule-1; IEC: Intestinal epithelial cells; IgA: immunoglobulin A; IL: interleukin; IFN: Interferon; IP-10: Interferon (IFN)-gamma-inducible protein-10; LFA-1: lymphocyte function-associated antigen-1; MAC-1: Macrophage-1 antigen; MAPK: mitogen activated protein kinase; MCP-1: macrophage chemoattractant protein; NF-κB: nuclear factor-kappa B; NO: Nitric oxide; PI3K: phosphoinositide-3 kinase; PLA_2_: phospholipase A_2_; PLC: phospholipase C; PGE_2_: prostaglandin E_2_; PKB: protein kinase B; PMA: phorbol-12 myristate-13 acetate; PPAR-γ: peroxisome proliferator-activated receptor gamma; ROS: reactive oxygen species; SCFAs: short chain fatty acids; SM: skeletal muscle; TNF-α: tumor necrosis factor-α; T reg: regulatory T cells; VCAM-1: vascular cell adhesion molecule-1.

## 1. Introduction

Human mucosal sites are colonized by an astonishing number of microorganisms (it is estimated that 90% of the cells in our body are microbes) of different kingdoms (e.g., fungi and bacteria) and genera (e.g., *Bifidobacterium*, *Eubacterium*, *Fusobacterium*, *Escherichia* and *Candida*), which are collectively referred to as microbiota or microflora. Most of these non-human cells are located in the gastrointestinal (GI) tract where they exert protective (*i.e.*, natural defense barrier and production of anti-microbial factors), structural (*i.e.*, development of immune system and induction of IgA) and metabolic (*i.e.*, fermentation of non-digestible dietary residues, synthesis of vitamins and ion absorption) functions [1]. 

**Figure 1:**
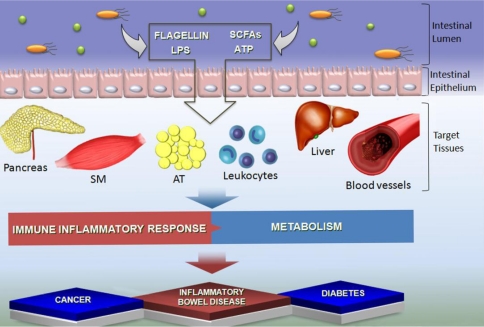
Schematic representation of the interaction between gut microbiota and host tissues. Soluble factors (e.g., LPS, flagellin, ATP and short chain fatty acids (SCFAs)) released by gut microbiota modulate host tissues such as pancreas, skeletal muscle (SM), adipose tissue (AT), leukocytes, liver and blood vessels function and may play a role in the development of several diseases including cancer, inflammation and diabetes.

A considerable advance in the understanding of the role of gut microbiota in both physiological and pathological conditions has been obtained. Important effects of these microorganisms and their products have been demonstrated not only in the GI tract but also in adipose tissue, immune and nervous systems [2,3,4]. Modifications in the proportions of microorganisms in the gut (qualitatively or quantitatively) and, consequently, in the concentrations of the compounds produced and released by them in the lumen have been suggested to play a role in the development of pathological conditions including inflammatory bowel disease (IBD), colon cancer, obesity and type 1 and 2 diabetes mellitus [3,4,5,6] (Figure 1). 

Some of the compounds that have been implicated in the effects of microbiota on host cells are microbial-derived ligands of toll like receptors (TLRs) such as LPS and flagellin, which activate, respectively, TLR-4 and -5 and modulate distinct aspects of host metabolism and immune responses [3,7]. Other compounds derived from the microbiota such as ATP also modulate the function of host tissues. This nucleotide induces the differentiation of an important subset of CD4+ lymphocytes, the Th17 cells [8]. Short chain fatty acids (SCFAs), which are the major metabolic products of anaerobic bacteria fermentation, have been suggested to be the link between microbiota and host tissues. The concentration of these fatty acids in the GI tract and blood may predispose to or prevents pathological conditions such as IBD, cancer and diabetes. Modifications in the concentrations or the ability of host tissues to use SCFAs have been described in these conditions [9,10,11,12,13].

Acetate (C_2_), propionate (C_3_) and butyrate (C_4_) are found in the human intestine at concentrations of approximately 13 mM in the terminal ileum, ~130 mM in the caecum and ~80 mM in the descending colon [14]. They are produced by anaerobic fermentation of non-digestible dietary residues and endogenous epithelial-derived mucus in the gut. SCFAs released in the intestinal lumen are readily absorbed and used as energy source by colonocytes (5 to 10% of human basal energy requirements are provided by SCFAs) and also by other tissues including liver and muscle [15]. SCFAs, of which butyrate is the most studied, modulate different processes including cell proliferation and differentiation, hormones secretion (e.g., leptin and peptide YY) [16,17] and activation of immune/inflammatory responses [4,18]. Therefore, in addition to energy supply, these fatty acids have other important functions. 

## 2. Effects of SCFA on Leukocyte Recruitment

Leukocytes are recruited and migrate from the bloodstream to the inflamed tissue in a multistep process that involves expression and activation of several proteins including adhesion molecules and chemokines and their coordinated interaction with endothelial cells [19]. Cell adhesion molecules such as selectins (E-, P- and L-selectins), integrins (e.g., lymphocyte function-associated antigen-1 (LFA-1) and macrophage antigen-1 (Mac-1)) and their counter ligands molecules (specific carbohydrates, vascular cell adhesion molecule-1 (VCAM-1), intercellular adhesion molecule-1 (ICAM-1) and others) play a central role in rolling, adhesion and transendothelial migration of leukocytes. Once in the extravascular space, leukocytes sense distinct sets of chemoattractants and move in the direction of the inflammatory foci [19].

SCFAs modify the recruitment of circulating leukocytes to the inflammatory site [4,20]. Interestingly, the effect of SCFAs on leukocytes varies depending on the experimental condition. Application of SCFAs in a sterile air pouch in the rat trunk induces a significant recruitment of leukocytes, particularly neutrophils [20]. However, oral administration of tributyrin, a pro-drug of butyrate, reduces migration of leukocytes to the peritoneum in response to inflammatory stimuli (oyster glycogen solution) [21]. This apparent discrepancy in the effects of SCFAs on leukocyte recruitment can be explained, at least in part, by their ability to act on different cells involved in the inflammation including neutrophils and macrophages, in which they act, respectively, inducing cell migration [20,22] and suppressing the production of pro-inflammatory cytokines [23]. This issue will be further discussed in the next sections of this review. 

### 2.1. Leukocyte Chemotaxis

**Figure 2:**
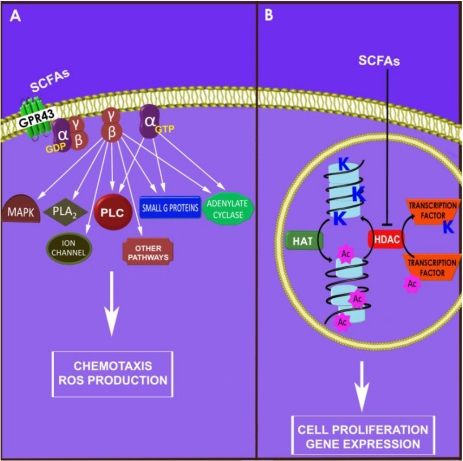
Schematic overview of the signaling pathways activated downstream of GPR43 receptors and representation of the effects of SCFAs through inhibition of histone deacetylase (HDAC) activity (**B**). GPR43 couples to Gi and Gq proteins, which interact with several proteins including adenylate cyclase, small G proteins (e.g., Rac and Rho), mitogen-activated protein kinases (MAPK), phospholipase C (PLC) and A_2_ (PLA_2_), ion channels and transcription factors (**A**). SCFAs may also act on cells through inhibition of HDAC (**B**). This class of enzymes, together with histone acetyltransferase (HAT), controls the acetylation state of histones and non-histone proteins and, consequently, modulates the transcription of several genes.

SCFAs induce directional migration (chemotaxis) of neutrophils *in vitro*, an effect that is dependent on the activation of a G protein-coupled receptor, the GPR43 [4,22,24,25]. This receptor was shown, as in the case of GPR41 and GPR109A, to be activated by SCFAs [26,27,28]. GPR43 couples to Gi/o and Gq proteins and is expressed in leukocytes, particularly neutrophils and monocytes, and in adipocytes [16,22,26,27]. Binding of agonists (SCFAs or synthetic agonists such as phenylacetamide-1 and -2) to this receptor activates several intracellular pathways including mitogen-activated protein kinases (MAPKs), protein kinase C and transcriptional factors such as activating transcriptional factor-2, also known as ATF-2 [4] (Figure 2(A)).

Evidence was obtained that SCFAs or phenylacetamide-1 elicit GPR43-dependent activation of PKB and MAPKs (p38 and ERK) in neutrophils. These responses were sensitive to pertussis toxin treatment, indicating a role for Gi proteins [25]. GPR43 agonists also induce rapid and transient activation of Rac1/2 GTPases and phosphorylation of ribosomal protein S6 in neutrophils. Genetic and pharmacological interventions that inhibit PI3Kγ, Rac2, p38 and ERK dramatically reduce the GPR43-dependent chemotaxis. These results are indicative of the participation of these signaling pathways in the movement of neutrophils in response to SCFAs [25].

Despite the fact that SCFAs clearly act as chemoattractant agents for neutrophils *in vitro*, the relevance of this interaction (SCFAs/GPR43) for the recruitment of leukocytes in the presence of other chemoattractants and molecules that regulate the activation of these cells is not clear. Evidence for an interaction between SCFAs/GPR43 and other chemottractant receptors has been provided [20,22,24]. Chemotaxis of neutrophils induced by formyl-Met-Leu-Phe (fMLP) or KC (CXCL1), an important chemokine for mouse neutrophils, has been shown to be either increased [24] or not affected by SCFAs [20,22]. SCFAs also reduce the surface expression of chemoattractant receptors (e.g., C5aR and CXCR2). This latter result is suggestive of an inhibitory effect of these fatty acids on neutrophil migration in response to other chemoattractants [4].

In contrast to what has been found in neutrophils, no effect of butyrate was observed in non- stimulated macrophage [29]. These authors have also found inhibition of LPS-induced migration of macrophages by butyrate [29]. This latter effect seems to be the result of inhibition of HDAC activity and, consequently, suppression of LPS-activation of Src [29], an enzyme that phosphorylates focal adhesion kinase (FAK), which has an important role in macrophage locomotion [30].

### 2.2. Chemokine Production

In addition to their chemotatic effect on neutrophils, SCFAs also modulate production and release of chemokines and expression of adhesion molecules in neutrophils [20] and endothelial cells [31,32,33], which may be relevant to the effect of these fatty acids on leukocyte recruitment. 

Chemokines attract and activate leukocytes. SCFAs modulate the production of these inflammatory mediators by neutrophils and other cells. Our group has shown that propionate and butyrate reduce the LPS-stimulated production of CINC-2 (also known as CXCL-2 and -3) by neutrophils [21] and by macrophages (data not published). This effect can explain, at least in part, the reduction of leukocyte recruitment observed in response to inflammatory stimuli after oral administration of tributyrin [21]. 

Cox *et al*. described the inhibitive effect that SCFAs, of which butyrate was the most and acetate the less potent, have on the production of macrophage chemoattractant protein-1 (MCP-1, also known as CCL2) either in the presence or absence of LPS [34]. This chemokine attracts monocytes/macrophages and lymphocytes. We have found that oral administration of butyrate reduces the production of MCP-1 by adipose tissue in diet-induced obese mice (data not published). MCP-1 plays an important role in macrophage recruitment and accumulation in the adipose tissue; an important factor for establishing insulin resistance in obese patients. 

IEC are source of cytokines and chemokines that regulate physiological and pathological processes in the GI tract. Because IEC are in contact with high concentrations of SCFAs, the effects of these fatty acids on these cells have been intensely investigated. Butyrate modulates the expression and release of IL-8, MCP-1 and growth-related oncogene (GRO, also known as CXCL-1) by IEC in response to cytokines and microbial-derived molecules such as peptideoglycan [35,36,37,38]. In human colonic subepithelial myofibroblasts, butyrate, but not acetate and propionate, blocks the expression of interferon (IFN)-gamma-inducible protein-10 (IP-10, CXCL-10), a chemoattractant for T lymphocytes and monocytes [39]. These effects that SCFAs have on chemokines may be relevant for the GI tract inflammatory and immune response. By changing the type or the amount of chemokines produced by intestinal cells, SCFAs may alter the recruitment of leukocytes and the pattern of inflammatory mediators produced in this tissue. 

### 2.3. Expression of Adhesion Molecules

SCFAs reduce the *in vitro* adherence of monocytes and lymphocytes to human umbilical vein endothelial cells (HUVEC) [31,32,40]. This effect is associated with an attenuation of NFκB and PPARγ activities and, consequently, suppression of adhesion molecules expression (ICAM-1 and VCAM-1) in endothelial cells. In monocytes, butyrate also reduces the constitutive and IFN-γ-induced expression of LFA-3 and ICAM-1 [41]. 

Butyrate or cholesteryl butyrate solid lipid nanoparticles have anti-adhesive effects on neutrophil interaction with HUVEC in the presence of fMLP or IL-1β [42]. Sina *et al*. found a significant reduction in L-selectin surface levels after incubation of neutrophils with propionate and butyrate [24]. We have found that acetate, propionate and butyrate do not affect the expression of β2 integrins in neutrophils, but increase that of L-selectin after 4 h of incubation [20]. However, the effects on L-selectin may not be relevant *in vivo*, since modifications in the expression of this adhesion molecule do not affect neutrophil rolling and adhesion [43]. Considering these findings, we hypothesize that SCFAs may affect neutrophil interaction with endothelial cells due to their effect on endothelial cells rather than on neutrophils.

## 3. Modulation of Leukocyte Effector Mechanisms by SCFAs

### 3.1. Production of Inflammatory Mediators by Immune Cells

Macrophages are the major source of inflammatory mediators involved in insulin resistance, atherosclerosis, rheumatoid arthritis and neurodegenerative diseases [44,45,46,47]. Once activated, macrophages produce large amounts of TNF-α, IL-1β and IL-6, chemokines, nitric oxide (NO) and arachidonic acid derivatives such as tromboxane A_2_, prostaglandins E_2_ and F_1α_.

SCFAs modulate the production of inflammatory mediators by macrophages as shown in Table 1. SCFAs, mainly butyrate, suppress the LPS- and cytokine-stimulated production of pro-inflammatory mediators including TNF-α, IL-6 and NO. Butyrate also enhances the release of the anti-inflammatory cytokine IL-10. However, this latter effect was not found in all studies; for example, Cox *et al*., instead of an increase, found an attenuation of IL-10 production by monocytes treated with SCFAs [34].

**Table 1:** Effect of SCFAs in the production of inflammatory mediators by isolated cells.

Cell type	Effect observed	Effective fatty acid	Reference
**Raw 264.7 cells**	↓ TNF-α, IL-6, NO, ↑ IL-10	Bt	[23,48]
**Mononuclear cells of the blood**	↓ TNF-α, ↑ PGE_2_	Bt	[49]
**Monocytes and macrophages**	↓ TNF-α	Bt	[50]
**Monocytes**	↓ TNF-α, IL-12, IFN-γ,	Bt	[51]
	↑ IL-10		
	↓ MCP-1, IL-10, ↑ PGE_2_	Ac, Pr and Bt	[34]
**Microglial cells**			
**-N9 cells**	↑ IL-6, NO	Pr and Bt	[52]
**-Rat primary microglia**	↓ TNF-α, IL-6, NO	Bt	[52]
**-Murine BV2 cell**	↓ NO	Bt	[53]
****Mesencephalic neuron-glia cultures****	↓ TNF-α, NO	Bt	[54]
**Kupffer cells**	↓ TNF-α, ↑ PGE_2_	Bt	[55]

Abbreviations: acetate (Ac), propionate (Pr), butyrate (Bt), interferon-γ (IFN-γ), interleukin-6 (IL-6), interleukin-10 (IL-10), interleukin-12 (IL-12), macrophage chemoattractant protein (MCP), nitric oxide (NO), prostaglandin E_2_ (PGE_2_), tumor necrosis factor-α (TNF-α). (↑) increase and (↓) reduction.

The main mechanism described for these effects is the attenuation of HDAC activity. Among the SCFAs, butyrate is the most potent, whereas acetate is the least potent inhibitor of HDAC [18,56]. This enzyme, together with the histone acetyltransferases (HAT), controls the degree of protein acetylation. By inhibiting the HDAC activity, SCFAs increase the acetylation of histone and non histone proteins including NFκB, MyoD, p53 and N-FAT [57] and, consequently, modulate gene expression (Figure 2(B)). 

The production of prostaglandin E_2_ (PGE_2_) is also modified by SCFAs. These fatty acids stimulated the *in vitro* production of this eicosanoid by human monocytes [58]. In accordance with this result, induction of PGE_2_ production was observed three hours after intraplantar injection of SCFAs and LPS in rat paws [34]. PGE_2_ has been considered an anti-inflammatory prostanoid due to its ability to attenuate the production of IL-1β and TNF-α by macrophages and Th1 differentiation. However, there is now evidence in favor of a pro-inflammatory action of this molecule [59]. PGE_2_, through activation of its receptor EP4, facilitates Th1 differentiation and Th17 expansion, two subsets of T helper involved in immune inflammation [59,60]. Considering these findings, SCFAs may also affect T cell differentiation. 

In addition to the classical eicosanoids, such as PGE_2_, other lipid mediators are also generated from polyunsaturated fatty acids including lipoxins, resolvins, protectins and maresins [61]. Despite their relevance to the resolution of the inflammatory process [61], at the moment, no study has been conducted in order to investigate the effect of SCFAs on the production of these lipid mediators. 

Anti-inflammatory actions of SCFAs have been also observed in neutrophils. Acetate, propionate and butyrate at 30 mM reduce TNF-α production by LPS-stimulated human neutrophils [62]. Propionate and butyrate inhibit the expression of pro-inflammatory mediators (TNF-α, CINC-2αβ and NO) in rat neutrophils, an effect that seems to involve attenuation of NF-κB activation [21].

Microglial cells are resident immune cells of the central nervous system (CNS). Activation of these cells leads to production of several inflammatory mediators (e.g., cytokines and NO) that participate in the defense reaction of the CNS against insults including microorganisms and damaged cells [63]. Chronic or excessive activation of these cells has detrimental effects on the CNS and seems to be involved in the initiation and progression of neurodegenerative diseases including Alzheimer and Parkinson’s disease. In spite of some controversy about the effect of SCFAs on microglial production of inflammatory mediators [52,53], most of the studies indicate that these fatty acids attenuate microglial activation, an effect that seems to involve HDAC inhibition [53,54]. These observations and the data obtained *in vivo* [64]support the proposition that SCFAs and other inhibitors of HDAC may be useful in preventing inflammation in the CNS. Indeed, Kim *et al*. [64] have shown that butyrate, valproic acid and trichostatin A (all inhibitors of HDAC activity) present antineuroinflammatory and neuroprotective effects in the ischemic brain of rats.

### 3.2. Effectors Mechanisms of Phagocytes

Once in the inflammatory site, neutrophils and macrophages internalize, kill and digest bacteria and fungi through mechanisms including production of reactive oxygen species (ROS) and release of granule enzymes. SCFAs affect the production of ROS and the phagocytic capacity of phagocytes [4,65,66,67]. This effect is important in the course of anaerobic bacteria infection. Both inhibition [65,66,68] and stimulation [4,68] of neutrophil phagocytosis by SCFAs have been described. In macrophages, butyrate reduce the phagocytic activity, an effect that probably arises from its inhibitory action on cell differentiation and maturation [69].

The effects of SCFAs on ROS production by neutrophils remain controversial. Some groups have found that SCFAs induce ROS production [4,70,71], whereas others have shown inhibition [65,67,72,73,74]. The discrepancy in the results obtained may be explained by differences in the protocols used such as the concentrations of SCFAs, measurement of ROS by using different methodologies (e.g., lucigenin**-**amplified chemiluminescence or reduction of cytochrome c), stimuli (e.g., PMA or fMLP), solution pH, source and state of neutrophil activation (e.g., neutrophils isolated from human blood or elicited rat neutrophils). 

### 3.3. Lymphocyte Activation and Response

Lymphocytes are involved in the adaptive immune response. These cells display membrane receptors that recognize a broad range of non-self antigens and allow them to generate specific responses to eliminate invading pathogens and infected or tumoral cells. SCFAs modify lymphocytes function as follows:

- T-cell proliferation: butyrate inhibits lymphocyte proliferation in response to several stimuli including concanavalin-A and immobilized anti-CD3 monoclonal antibody [41,75].
- Production of cytokines: incubation of lymphocytes with butyrate reduces the production of interleukin-2; this cytokine stimulates growth, differentiation and survival of antigen-selected T-lymphocytes, and interferon-γ (IFN-γ) after stimulation with concanavalin-A or anti-CD3 and anti-CD8 [76,77]. This latter cytokine is particularly important in response to viral infection, tumor cells and in auto-immune conditions. On the other hand, butyrate presents an opposite effect on the production of IL-10 by lymphocytes [75].
- Production of regulatory T (T_reg_) cells: this subpopulation of T cells actively suppresses immune function and is considered an attractive target for the treatment of immunological and inflammatory pathologies. HDAC inhibitors enhance the production and suppressive function of regulatory T cells [77]. Considering that SCFAs, as previously described, also suppress the activity of HDAC, we hypothesize that these fatty acids may also exert their effects on inflammation and immune responses through regulation of this subset of T cells.

## 4. Therapeutical Application of SCFAs in Inflammatory Conditions

Several studies have attempted to use SCFAs or their derivatives therapeutically in inflammatory conditions *in vivo*, in both human and animal models. Some of these studies are summarized in Table 2. 

A large number of studies in which SCFAs were provided by different forms including ingestion of dietary fiber, use of enemas or oral administration of sodium butyrate were performed to investigate their effects on IBD. Although SCFAs or compounds (dietary fibers) that increase the availability of these fatty acids present beneficial effects in intestinal inflammation, this positive effect is not consensual. This discrepancy may be due to the use of distinct experimental protocols. Further studies are necessary to establish the dose, frequency and form of SCFAs administration in order to optimize results. Other aspects, such as stage of disease and possibility of association with other drugs, must also be investigated in more detail.

**Table 2:** Effects of SCFAs or their derivatives in inflammatory conditions.

Disease	Route of administration and dosage	Effect	Reference
**Inflammatory bowel disease**	Diet with RS (1.53 kg/10 kg of diet)	Improvement of symptoms; epithelial cell proliferation; regeneration of laminin; growth of intestinal bacteria	[78]
	Diet supplemented with cellobiose (9%)	Reduction of weight loss; diminished tissue edema; attenuation of inflammatory cytokine concentrations	[79]
	Fiber supplementation (5%) before and after TNBS colitis	Reduction in MPO and NO synthase activities; restoration of colonic glutathione levels; diminished TNF-α concentrations	[80]
	SB enemas (100 mM)	Increased the duration of pain in rats with colitis	[81]
		Improves clinical symptoms and inflammatory scores	[82]
	SB enemas (100 mM)	Minor effects on colonic inflammation and oxidative stress; increased IL-10/IL-12 ratio and CCL5 concentrations	[83]
	5-ASA (2 g) + SB (80 mM) enemas	Improvement versus the baseline; only one remission	[84]
	Oral SB (10 mg/kg)	Improvement of mucosa lesion and attenuation of the inflammatory profile of intestinal mucosa and local lymph nodes in a model of DSS-induced colitis	[85]
**Sepsis and ALI**	SB (500 mg/kg) intravenous (i.v.) injection	Reduced serum alanine aminotransferase, MPO activity and creatinine concentrations; improved survival rates	[86]
	Oral butyrate (10 mg/kg)	Attenuation of lung histopathological changes, alveolar hemorrhage and neutrophil infiltration	[87]
	TSA (2 mg/kg) or SB (200 mg/kg) intraperitoneal (i.p.) injection	Reduced neutrophil infiltration, inhibited ICAM-1 and E-selectin expression in lung	[88]
**Ischemia induced injury**	SB (100 or 300 mg/kg, i.p.)	Reduced infarct size	[89]
		Diminished brain infarct volume and microglial activation	[64]

Abbreviations: Acute lung injury (ALI), Aminosalicylic Acid (ASA), Intercellular Adhesion Molecule (ICAM-1), Interleukin (IL), Nitric oxide (NO), Mieloperoxidase (MPO), Resistant Starch (RS), Short chain fatty acids (SCFA), Trichostatin (TSA), Trinitrobenzenesulfonic acid (TNBS), Tumor Necrosis Factor-α (TNF-α).

Sepsis is a condition defined by the presence of both infection and an inflammatory systemic response. In this condition there is an excessive production of inflammatory mediators such as TNF-α, IL-1 and high-mobility group box (HMGB1). These mediators cause tissue injury and multiple-organ dysfunction. Despite significant advances in sepsis management, severe sepsis (sepsis with organ dysfunction) and septic shock (a condition characterized by the presence of a hypotension despite adequate volume resuscitation, in the absence of other causes for hypotension) still present mortality rates of 25 to 30% and 40 to 70%, respectively. Some authors tested whether butyrate could have beneficial effects in this condition [86,87,90]. In a model of sepsis induced by caecal ligation and puncture (CLP) in rats, sodium butyrate administration prevented damage to the liver, kidneys and lungs, and improved the survival rate [86]. The authors associated these effects with a reduction in the production of HMGB1. This mediator is secreted by activated monocytes, macrophages and neutrophils and triggers the release of several pro-inflammatory cytokines (e.g., TNF-α and IL-1) [91] and plays a key role for the establishment of severe sepsis [92].

Promising results were obtained with the use of butyrate for reducing lung injury caused by sepsis [87,93]. Acute lung injury (ALI) is characterized by loss of the alveolar-capillary barrier integrity and refractory hypoxemia [94]. Sepsis is the most usual cause of ALI [95]. The pretreatment with sodium butyrate significantly alleviated septic lung injury and attenuated histological lesions in mice [93]. Reductions of leukocyte infiltration and pulmonary myeloperoxidase (MPO) activity were observed in mice treated with butyrate. Such effects have been associated to inhibition of HDAC activity [93].

In another study in which ALI was induced by intratracheally instillation of LPS in mice, oral administration of butyrate (10 mg/kg) reduced the concentrations of TNF-α, IL-1β and nitric oxide (NO) in bronchoalveolar lavage fluid [87]. Reduction in alveolar hemorrhage and infiltration of neutrophils were also observed indicating a protective effect of butyrate [87]. 

The effect of sodium butyrate and other inhibitors of HDAC (valproic acid and trichostatin A) was also investigated on tissue injury induced by ischemia [64,89]. Hu *et al*. found that butyrate pretreatment reduced the infarct size in a model of myocardial ischemia and reperfusion in rats. This beneficial effect of butyrate was attributed to the suppression of HMGB1 production [89]. In rats submitted to cerebral artery occlusion, post-insult treatment with butyrate reduced brain infarct volume and suppressed microglial activation [64]. According to the authors, multiple mechanisms are involved in stroke, and due to the fact that there is no effective treatment for this disease, butyrate should be tested in clinical trials.

## 5. Conclusions

SCFAs present multiple effects in different cells involved in the inflammatory and immune responses. These fatty acids not only affect the function of leukocytes (e.g., production of inflammatory mediators and ability of leukocytes to migrate) but can also induce apoptosis in lymphocytes [96,97], macrophages [98] and neutrophils [99]. The latter effect may be relevant for the outcome of the inflammatory process and the immune response to bacteria that produce these fatty acids. 

In general, SCFAs, such as propionate and butyrate, inhibit stimuli-induced expression of adhesion molecules, chemokine production and consequently suppress monocyte/macrophage and neutrophil recruitment, suggesting an anti-inflammatory action. However, there is also evidence in favor of a pro-inflammatory action of SCFAs in some conditions [20,100]. This discrepancy may be in part explained by the ability of SCFAs to induce neutrophil migration. In sites of anaerobic bacteria infection or after loss of intestinal epithelial integrity, high concentrations of SCFAs may lead to neutrophil accumulation and amplification of the inflammatory process. Another possible explanation is the fact that these fatty acids may present divergent effects depending on the cell type (e.g., anti- and pro-inflammatory effects of SCFAs on macrophage and microglial cells have been demonstrated [52,97,101]). Therefore, although SCFAs modulate the function of immune cells, more studies are necessary in order to understand the precise role of SCFAs on the interaction between bacteria and host immune cells *in vivo*, particularly in the GI tract and in sites of anaerobic infections including the skin, oral cavity and respiratory tract.
